# Whole-genome sequencing investigation of animal-skin-drum-associated UK anthrax cases reveals evidence of mixed populations and relatedness to a US case

**DOI:** 10.1099/mgen.0.000039

**Published:** 2015-11-07

**Authors:** Steven T. Pullan, Talima R. Pearson, Jennie Latham, Joanne Mason, Barry Atkinson, Nigel J. Silman, Chung K. Marston, Jason W. Sahl, Dawn Birdsell, Alex R. Hoffmaster, Paul Keim, Richard Vipond

**Affiliations:** ^1^​ Public Health England, Microbiology Services, Porton Down, Salisbury, Wiltshire, UK; ^2^​ Center for Microbial Genetics and Genomics, Northern Arizona University, Flagstaff, AZ, USA; ^3^​ Bacterial Special Pathogens Branch, Division of High-Consequence Pathogens and Pathology, Centers for Disease Control and Prevention, Atlanta, GA, USA

**Keywords:** animal-hide, anthrax, phylogeography, UK

## Abstract

There have been two anthrax cases affecting people that played and/or made animal-skin drums in the UK during the last 10 years, with single fatal occurrences in Scotland in 2006 and London in 2008. Investigations by the Health Protection Agency (now Public Health England) employing multi-locus-variable number tandem repeat analysis had previously linked the clinical cases to spores associated with animal skins and drums the patients had been in contact with. In this study, whole-genome sequencing of 23 *Bacillus anthracis* isolates harvested during the investigations was performed. High-quality draft assemblies of these genomes provided greater characterization of the *B. anthracis* strains present and placed them all upon a new branch of the global phylogeny. Although closely related, the clinical isolates from the two events, and another isolated from a drum-skin-associated case in New York in 2006, were distinct from each other. Multiple distinct genotypes were found during both investigations, implying either multiple contamination events or a single heterogeneous contamination. One environmental isolate from the Scottish incident was more closely related to London isolates than to the other Scottish isolates. As *B. anthracis* of this subgroup was present at both geographically and temporally distinct events, it may be more widespread at the source of contamination. All isolates were distinct from currently characterized West African strains, despite this being the likely origin of the drums and hides, therefore adding to our knowledge of *B. anthracis* diversity in the region.

## Data Summary

1. FASTQ data for all samples has been deposited in the Sequence Read Archive under BioProject accession PRJNA287512 (http://www.ncbi.nlm.nih.gov/sra/?term = PRJNA287512)
2. Table S4 has been deposited in Figshare: http://dx.doi.org/10.6084/m9.figshare.1569313

## Impact Statement

This paper describes whole-genome sequencing (WGS) of 23 *Bacillus anthracis* isolates collected during public health investigations into two fatal anthrax cases involving animal-skin-drum enthusiasts in 2006 and 2008 in the UK. The data confirm, at a level of resolution far greater than previously achieved, the link between the clinical cases and the contaminated drums and skins that the deceased individuals had been in contact with. WGS revealed that the incidents involved related but distinct strains and allowed us to phylogenetically place the two sets of strains together with an isolate from a similar US case, on a novel branch of the global *B. anthracis* phylogeny that is distinct from existing characterized West African isolates, despite this being the source of the skins in question. This information adds to the fundamental understanding of *B. anthracis* phylogeography and is potentially of value in the source attribution of anthrax outbreaks which may occur from one of an increasing number of routes of exposure and infection observed in the last decade.

## Introduction

Human cases of anthrax, caused by the Gram-positive endospore-forming bacterium *Bacillus anthracis*, have historically often been associated with the industrial processing of animal hides (CDC, 2006; Osborn, 1920). However, there have been a number of anthrax cases in the USA and UK associated with the recreational use of drums that were skinned using hides imported from West Africa. In the USA, anthrax cases occurred in New York in 2006 (CDC, 2006; Nguyen *et al.*, 2010), Connecticut in 2007 (Guh *et al.*, 2010) and New Hampshire in 2009 (CDC, 2010). Four individuals were infected: two were drum makers, one a relative of the drum maker and one simply played drums. Genotyping (Marston *et al.*, 2011) determined that all isolates belonged to the A.Br.011/009 canonical SNP (canSNP) lineage (Van Ert *et al.*, 2007). In the UK, there have been two cases of inhalational anthrax associated with West African drum skins. In Scotland, in 2006, a 50-year-old man died from a non-specific flu-like illness that was retrospectively diagnosed as anthrax (Riley, 2007). In London, in 2008, a 34-year-old man also suffered a fatal case of anthrax (Anaraki *et al.*, 2008). In both cases the victims were known to have interests in the playing and making of Djembe drums, and had access to hides and drums thought to have been imported from West Africa [Guinea in the 2006 case (Riley, 2007); multiple locations, including The Gambia in the 2008 case (Anaraki *et al.*, 2008)]. Contemporaneous investigations isolated *B. anthracis* from drums, hides and storage areas that matched the genotype of the clinical isolates, as determined by multi-locus variable number tandem repeat analysis (MLVA-8) (Keim *et al.* 2000; Public Health England, internal communications). In neither case was an individual drum or skin identified as the source, as *B. anthracis* was harvested from multiple items. In addition, in each case an environmental isolate was found with an alternative MLVA-8 profile to that of the clinical isolates, demonstrating that a diverse genotypic population of spores was present at each site. The aim of this study was to employ whole-genome sequencing (WGS), which has revolutionized the study of disease outbreaks in the intervening years since the events described for a number of pathogens (Eyre *et al.*, 2013; Gardy *et al.*, 2011; Köser *et al.*, 2012) including *B. anthracis* (Derzelle *et al.*, 2015; Price *et al.*, 2012), to further characterize all isolates collected from both UK events, to place them in the global anthrax phylogeography and to better estimate the level of population variation present.

## Methods

### *B. anthracis* isolates

Isolates were recultured from the Public Health England anthrax strain collection glycerol stocks. A 10 μl loop of glycerol suspension was streaked onto Columbia agar plus 5 % horse blood (bioMérieux) and incubated aerobically for 16–24 h at 37 °C.

### DNA extraction

DNA was harvested from a single colony suspended in 500 μl ATL buffer using a QIAamp UCP Pathogen Mini kit (Qiagen) according to manufacturer's instructions and including the mechanical pre-lysis protocol.

### MLVA-8

MLVA-8 (Keim *et al.*, 2000) using the modified HCvrrC1 and HCvrrC2 loci primers (Stratilo & Bader, 2007) was used. Briefly, 23 μl molecular-grade water, 1 μl each primer (10 μM) and 1 μl genomic DNA (50–100 ng) were added to PCR beads (GE Healthcare). Samples were heated to 95 °C for 5 min, followed by 35 cycles of 30 s at 95 °C, 30 s at 56 °C and 30 s at 72 °C, with a final extension at 72 °C for 7 min. PCR products were purified using a DyeEx 2.0 spin kit (Qiagen) and concentrated (Concentrator Plus; Eppendorf) following the manufacturer's protocols. Samples were resuspended in 0.5 μl GeneScan-500 ROX size standard (Applied Biosystems) with the addition of 19.5 μl Hi-Di Formamide. Product length was determined using a 3130xl genetic analyser and analysed with GeneMapper software (Applied Biosystems). Estimated fragment sizes were corrected to the nearest exact actual size reported (Keim *et al.*, 2000; Stratilo & Bader, 2007).

### CanSNP typing

CanSNP analysis was performed as described previously (Van Ert *et al.*, 2007). A 10 μl total reaction volume comprised of 1 ×  TaqMan Universal PCR Mastermix (Applied Biosystems), 250 nM each probe, 600 nM each primer, 1 μl DNA template (50–100 ng) and 3 μl molecular-grade water. Samples were heated in a thermocycler at 50 °C for 2 min and 95 °C for 10 min followed by 40 cycles of 95 °C for 15 s and 60 °C for 1 min. End-point fluorescence data were measured using an ABI 7900 real-time platform (Applied Biosystems).

### WGS

Purified genomic DNA was sequenced by Public Health England Genomic Services and Development Unit. DNA was tagged and multiplexed with a Nextera XT DNA kit (Illumina) and sequenced on an Illumina Hi-Seq 2500 platform with paired-end read lengths of 150 bp. A minimum 150 Mb of Q30-quality data was obtained for each isolate.

### Data analysis

Sequencing reads were trimmed to remove adaptors and low-quality bases, with quality checked using Trimmomatic (Bolger *et al.*, 2014) and FastQC (http://www.bioinformatics.bbsrc.ac.uk/projects/fastqc). Reads were then mapped to the ‘Ames Ancestor’ chromosomal sequence (GenBank accession number NC_007530.2), as well as pX01 and pX02 sequences (GenBank accession numbers NC_007322.2 and NC_007323.3) using bwa 0.7.5 (Li & Durbin, 2009). The SAM output file was converted to BAM format using SAMtools (Li *et al.*, 2009). Unified Genotyper 0.0.7 from gatk2 (DePristo *et al.*, 2011) was used to call bases and create a VCF file for each sample, which was further filtered to extract core genome SNPs that passed the filtering criteria of mapping quality >30, variant ratio >0.9, read depth >5 and ratio of reads with MQ0 to total number of reads < 0.05. Polymorphic positions were included in a multiple sequence alignment of all samples. All of the above were performed on the Public Health England Galaxy instance (Blankenberg *et al.*, 2010; Giardine *et al.*, 2005; Goecks *et al.*, 2010). SNP filtering criteria were validated using simulated data generated using ArtificialFastQGenerator (Frampton & Houlston, 2012). Maximum-parsimony phylogeny was inferred in mega6 (Tamura *et al.*, 2013). FigTree 1.4.2 (http://tree.bio.ed.ac.uk/software/figtree) was used to produce all final figures. SPAdes 3.1.1 (Bankevich *et al.*, 2012) was used to produce draft assemblies for each isolate. Harvest suite (Treangen *et al.*, 2014) was used to generate a core genome alignment including publicly available sequence data listed in Table S3 (available in the online Supplementary Material) and export core genome SNPs that passed quality filters to a multiFASTA file used as input for mega. FASTQ data were deposited in the Short Read Archive under accession PRJNA287512 [Data Citation 1].

## Results and Discussion

### MLVA-8

MLVA-8 analysis performed during the Health Protection Agency (now Public Health England) investigation into the cases, at the times they occurred (Table S1, available in the online Supplementary Material), suggested that there were three distinct profiles discovered in isolates collected during the Scottish investigation. A common profile was shared by the clinical samples and all but three of the environmental samples (profile M1). The three other environmental samples consisted of one isolate, Scotland_474, with a profile that differed at the *VrrA* locus (profile M2), and two isolates with genotypic profiles that differed from the majority profile at the pX01 locus. Caution was taken in the interpretation of the latter, as the repeat size was small and the product size estimation method used was susceptible to inaccuracy in this range. The WGS performed in this study allowed the confirmation of repeat size at this short locus. Extraction of reads spanning across the region demonstrated that in fact all isolates had an equal repeat length at the loci, therefore reducing the number for MLVA-8 genotypes present to two.

The London isolates all shared a common MLVA-8 profile (profile M3), with the exception of London_496, which had alternative lengths at the *vrrA* and *vrrC1* loci (profile M4). Both were distinct from Scottish isolate profiles.

In both cases, the clinical isolates matched the most common environmental profile and these results were used to conclude in both investigations that the anthrax cases were contracted from association with animal skins or drums.

### CanSNP profiling

Van Ert *et al.* (2007) identified 13 canSNPs that describe the three major lineages and 12 clonal sublineages of *B. anthracis*. All animal-skin-drum-associated strains isolated in the USA belong to the A.Br.008/009 group, as defined by the 13 canSNPs, and have been further classified with the addition of novel SNP assays as belonging to the A.Br.011/009 subgroup (Marston *et al.*, 2011).

CanSNP analysis of the UK isolates showed that all have the A.Br.008/009 group canSNP profile identical to the USA isolates. *In silico* extraction of the A.Br.011 and A.Br011/009_3692595 SNP loci from the WGS also showed agreement with the USA isolates and placed all UK isolates into the A.Br.011/009 group data (in agreement when measured by either mapping to the Ames Ancestor reference or through *de novo* assembly and alignment). In both the USA and UK cases, the skins involved were thought to have been imported from West Africa. Agreement in their canSNP profiles reflects their likely shared geographical origin.

### WGS

WGS was performed to further characterize the diversity present within the UK isolates. Sequence reads were mapped to the Ames Ancestor genomic sequence, as well as to the pX01 and pX02 plasmid sequences. All samples provided at least 97 % chromosomal sequence coverage at a read depth of ≥ 10 (mean coverage varying between 39 ×  and 96 × ; Table S3).

All 15 Scottish isolates and six of the nine London isolates mapped to both plasmid sequences with >99 % coverage at a read depth of ≥ 10, confirming them as true *B. anthracis* isolates (mean coverage varying between 98 ×  and 535 × ), but three of the nine London samples showed insignificant mapping to pX02. Contemporaneous MLVA-8 analysis of the outbreak had recorded a positive signal for the pX02 locus, as had the pX02-targeted capsule PCR test (Public Health England, result not shown). It is therefore likely that the pX02 plasmid has been cured during storage in glycerol or during the latest culture of these isolates. For comparison, Centers for Disease Control and Prevention sequence data from an isolate from the 2006 New York case (CDC, 2006; Nguyen *et al.*, 2010) was also included in the analysis.

### Whole-genome SNP analysis

**Fig. 1.**
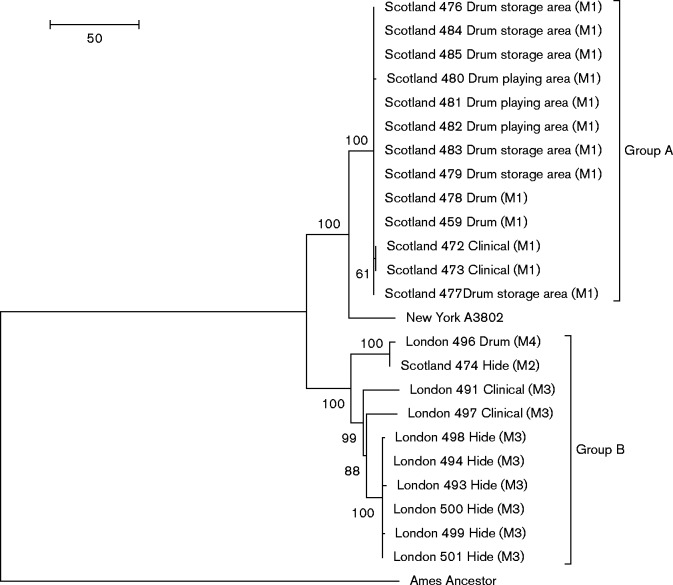
Maximum-parsimony phylogeny of the drum-skin-associated isolates. Reconstructed in mega6 (Tamura *et al.*, 2013) using the spr algorithm, with 572 SNPs identified by WGS of the 24 isolates using Ames Ancestor as reference. Bootstrap values (1000 replicates) are shown, but likely underestimate confidence in clonal populations with little homoplasy (Pearson *et al.*, 2009). Branch length in units of number of changes over the whole sequence. There are no homoplasies in the topology. Location and sample source are shown for each isolate, MLVA-8 profiles are indicated in parentheses.

SNPs meeting strict mapping and quality criteria (see Methods for details) were identified in each sample using the Ames Ancestor sequence as reference. In total, 572 SNPs were found within the isolates and these positions were used to infer a maximum-parsimony phylogeny, shown in Fig. 1. The UK strains fall into two groups: Group A consists solely of strains isolated during the Scottish case, whilst Group B contains all London case strains and a single Scottish strain. The two separate clades are well supported; with 38 synapomorphic SNPs separating Group A and 25 Group B from their most recent common ancestor (a synapomorphic SNP is a genome position that has mutated such that the new nucleotide is shared with all descendants). The phylogeny is robust and contains no homoplasies. Group A is the less diverse of the two groups, containing just three genotypes; a genotypic group of 10 strains, a genotypic pair defined by a single synapomorphic SNP and a unique genotype defined by a single autapomorphic SNP (an autapomorphic SNP is a genome position that has mutated but is found only in a single descendant). Group B is more diverse, containing eight genotypes, with 84 SNPs represented within the group, with only two genotypic pairs within the 10 strains. The subgroup of Group B, containing the London_496 and Scotland_474 strains, is defined by 22 synapomorphic SNPS.

### Scottish isolates

MLVA-8 analysis performed at the time of the 2006 case indicated that three genotypes were present amongst the isolated strains. Correction of the pX01 locus lengths using the WGS data reduced this to just two: a predominant profile (M1) describing the clinical isolates and the majority of environmental isolates, whilst a second profile (M2) was found in a single strain (Scotland_474) that differed only at the *vrrA* locus (Table S1). The WGS data presented here clearly support the distinct genotype attributed to Scotland_474, as it is the only Scottish isolate to fall outside of Group A in the phylogeny (Fig. 1). All other isolates that shared the M1 profile are closely related, within a maximum distance of two SNPs. The clinical isolates Scotland_472 and Scotland_473 differ from all other Group A members by a single shared SNP, located within the GBAA_5087 gene, encoding an OmpR family DNA-binding response regulator. The presence of this SNP within duplicate patient isolates makes it unlikely to have arisen during culture for analysis. Selection of this mutation within the host, or presence of this variant within the population prior to infection are more likely. This SNP is not found in any other publicly available genomes (listed in Table S2). The level of relatedness suggests that all Group A strains had a common source. As they were isolated from several different drums, as well as multiple areas in which the drums had been stored or played, it is most likely that cross-contamination from a single initial contaminated item occurred. The presence of the phylogenetically distinct Scotland_474 strain, on a hide that did not test positive for any other strains, implies that a separate contamination event introduced this strain to the environment.

### London isolates

The London isolates shared a common MLVA-8 profile (M3) with the exception of London_496, which had alternative lengths at the *vrrA* and *vrrC1* loci (M4) (Table S1). The whole-genome SNP-based phylogeny also places this isolate as an outlier, falling within Group B, but within a subgroup along with Scotland_474. The presence of these two closely related strains during the geographically distinct cases implies that strains belonging to this subgroup are a more common contaminant of hides.

The clinical isolate London_491 has an MLVA-8 profile identical to all environmental isolates other than London_496. However, WGS reveals a wider range of diversity within the isolates and places London_491 on its own branch, defined by 20 autapomorphic SNPs. The level of diversity seen within the London isolates is much greater than that seen in the Group A isolates. It is far beyond what is credible to have arisen during culture for analysis (Vogler *et al.*, 2002; Ågren *et al.*, 2014). It is more likely to represent contamination by a heterogeneous population through multiple contamination events.

This increased analytical resolution highlights the discriminatory potential of the whole-genome approach when compared to traditional molecular analysis alone. In addition, prior to the application of WGS, the level of relatedness of Scotland_474 and London_496 and their link to Group B was unclear, as their MLVA-8 shares more in common with Group A.

### New York isolate

The isolate taken from the New York 2006 case shares a more recent common ancestor to the Group A strains, but is differentiated from them by 26 synapomorphic SNPs.

### Phylogeography

**Fig. 2.**
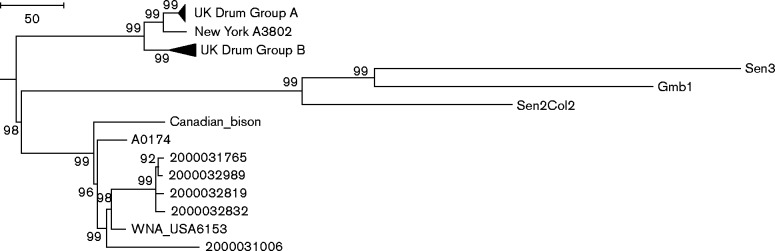
Subtree of a core genome SNP maximum-parsimony phylogeny of global *B. anthracis* isolates. Core genome SNPs were identified using Harvest suite (Treangen *et al.*, 2014) from the strains listed in Table S1. Phylogeny reconstructed in mega6 (Tamura *et al.*, 2013) using the spr algorithm, with 11 392 SNPs identified across 124 isolates Consistency Index (CI = 0.975). The full tree is included as Fig. S1. Bootstrap values (1000 replicates) are shown, but likely underestimate confidence in clonal populations with little homoplasy (Pearson *et al.*, 2009). Groups A and B refer to the groups defined in Fig. 1. Sequence accession numbers are shown in Table S3.

In all of the UK and USA drum-related infections, the skins involved were thought to have been imported from West Africa (Anaraki *et al.*, 2008; Marston *et al.*, 2011; Riley, 2007). Recently, the genome sequences of three West African strains, i.e. two from Senegal and one from Gambia, have been reported (Rouli *et al.*, 2014). These, along with *de novo* assemblies of the drum strains and the entire collection of *B. anthracis* assemblies present in GenBank (listed in Table S1) were aligned and core genome SNPs identified using Harvest suite tools (Treangen *et al.*, 2014). The exported SNPs were then used to infer a maximum-parsimony phylogeny in mega6 (Tamura *et al.*, 2013). The full tree is shown in Fig. S1. The drum-skin-related isolates form a novel clade, nested within a larger clade containing the West African strains as well as Canadian and US strains; Fig. 2 shows this subtree. The drum-skin-specific clade is defined by 101 shared SNPs, specific to this branch, and the West African strains clade defined by 222. The branch leading to the putative ancestor of both the West Africa and western North America clades is defined by four SNPs, which are not present in any other of the 124 analysed sequences (positions 1 776 654, 2 917 555, 4 544 130 and 4 544131; full SNP data for all strains can be found in supplementary files). The consistency index for the full phylogeny is 97.5 %. There is only one homoplastic SNP between the existing West African strains clade and the new Drum isolates, and this is only present in a single drum strain of 23 and a single West African isolate. There are 17 homoplasious sites shared by Ames Ancestor and at least one of the existing West African strains, but not by any of the A.Br.008/009 lineage strains placed between them, none of which affect the placement of the novel drum isolates branch. There is strong support therefore for the isolates forming a novel branch, distinct from the existing West African strains.

The strains associated with the two UK and one New York cases are more closely related to each other than they are to any previously published sequences. Furthermore, no epidemiologically unrelated samples in >1300 genotyped geographically diverse isolates from the Northern Arizona University *B. anthracis* collection fall into this novel West African clade (P. Keim, D. Birdsell and T. R. Pearson unpublished data). This reflects their likely shared geographical origins as all hides were thought to have been imported from West Africa. The fact that they are distinct from the West African strains sequenced to date is also not surprising as the number of sequences available is low and thus not likely to be representative for such a large geographical region. The skins and hides present in the New York incident were known to have been imported from Côte d'Ivoire (CDC, 2006) and those from the Scottish incident were thought to have been imported from Guinea (Riley, 2007) which are not represented amongst currently available sequences. The multiple skins associated with the London incident were thought to be of mixed origin including The Gambia (Anaraki *et al.*, 2008), but show no significantly closer relationship to the published Gambian sequence. These strains therefore increase our knowledge of global *B. anthracis* phylogeography.

## Conclusions

The two UK animal-skin-drum-related anthrax cases were caused by independent importation of animal hides contaminated with *B. anthracis* strains belonging to the same lineage as that responsible for drum-skin-related anthrax cases in New York. They also belong to the same canSNP group as other US cases for which whole-genome data are unavailable. The two fatalities were caused by distinct strains, but a pair of closely related isolates was found between the two sites, implicating them as a common presence in contaminated hides. The use of WGS has increased our understanding of the populations of anthrax spores present at each scene and given us new insight into the relationship between the two events as well as their relatedness to a US case. The strains isolated and sequenced have added to the global *B. anthracis* phylogeographic profile.

## Supplementary Data

282 Supplementary Data

## References

[mgen000039-AnarakiAnaraki1] Anaraki S. Addiman S. Nixon G. Krahé D. Ghosh R. Brooks T. Lloyd G. Spencer R. Walsh A. other authors (2008). Investigations and control measures following a case of inhalation anthrax in East London in a drum maker and drummer, October 2008 Euro Surveill 13 19076. 19094916

[mgen000039-agren1] Ågren J. Finn M. Bengtsson B. Segerman B. (2014). Microevolution during an Anthrax outbreak leading to clonal heterogeneity and penicillin resistance PLoS One 9 e89112 10.1371/journal.pone.0089112. 24551231 PMC3923885

[mgen000039-Bankevich1] Bankevich A. Nurk S. Antipov D. Gurevich A. A. Dvorkin M. Kulikov A. S. Lesin V. M. Nikolenko S. I. Pham S. other authors (2012). SPAdes: a new genome assembly algorithm and its applications to single-cell sequencing J Comput Biol 19 455–477 10.1089/cmb.2012.0021 . 22506599 PMC3342519

[mgen000039-Blankenberg1] Blankenberg D. Von Kuster G. Coraor N. Ananda G. Lazarus R. Mangan M. Nekrutenko A. Taylor J. (2010). Galaxy: a web-based genome analysis tool for experimentalists Curr Prot Mol Biol 19 101–1021. 10.1002/0471142727.mb1910s89 PMC4264107 20069535

[mgen000039-Bolger1] Bolger A. M. Lohse M. Usadel B. (2014). Trimmomatic: a flexible trimmer for Illumina sequence data Bioinformatics 30 2114–2120 10.1093/bioinformatics/btu170 . 24695404 PMC4103590

[mgen000039-CDC1] CDC (2006). Inhalation anthrax associated with dried animal hides- Pennsylvania and New York City, 2006 MMWR Morb Mortal Wkly Rep 55 280–282. 16543883

[mgen000039-CDC12] CDC (2010). Gastrointestinal anthrax after an animal-hide drumming event-New Hampshire and Massachusetts, 2009 MMWR Morb Mortal Wkly Rep 59 872–877. 20651643

[mgen000039-DePristo1] DePristo M. A. Banks E. Poplin R. Garimella K. V. Maguire J. R. Hartl C. Philippakis A. A. del Angel G. Rivas M. A. other authors (2011). A framework for variation discovery and genotyping using next-generation DNA sequencing data Nat Genet 43 491–498 10.1038/ng.806 . 21478889 PMC3083463

[mgen000039-Derzelle1] Derzelle S. Girault G. Roest H. I. J. Koene M. (2015). Molecular diversity of *Bacillus anthracis* in the Netherlands: investigating the relationship to the worldwide population using whole-genome SNP discovery Infect Genet Evol 32 370–376. 25843650 10.1016/j.meegid.2015.03.030

[mgen000039-Eyre1] Eyre D. W. Cule M. L. Wilson D. J. Griffiths D. Vaughan A. O'Connor L. Ip C. L. C. Golubchik T. Batty E. M. other authors (2013). Diverse sources of *C. difficile* infection identified on whole-genome sequencing N Engl J Med 369 1195–1205 10.1056/NEJMoa1216064 . 24066741 PMC3868928

[mgen000039-Frampton1] Frampton M. Houlston R. (2012). Generation of artificial FASTQ files to evaluate the performance of next-generation sequencing pipelines PLoS One 7 e49110 10.1371/journal.pone.0049110 . 23152858 PMC3495771

[mgen000039-Gardy1] Gardy J. L. Johnston J. C. Ho Sui S. J. Cook V. J. Shah L. Brodkin E. Rempel S. Moore R. Zhao Y. other authors (2011). Whole-genome sequencing and social-network analysis of a tuberculosis outbreak N Engl J Med 364 730–739 10.1056/NEJMoa1003176 . 21345102

[mgen000039-Giardine1] Giardine B. Riemer C. Hardison R. C. Burhans R. Elnitski L. Shah P. Zhang Y. Blankenberg D. Albert I. other authors (2005). Galaxy: a platform for interactive large-scale genome analysis Genome Res 15 1451–1455 10.1101/gr.4086505 . 16169926 PMC1240089

[mgen000039-Goecks1] Goecks J. Nekrutenko A. Taylor J. Galaxy Team T. Galaxy Team (2010). Galaxy: a comprehensive approach for supporting accessible, reproducible, and transparent computational research in the life sciences Genome Biol 11 R86 10.1186/gb-2010-11-8-r86 . 20738864 PMC2945788

[mgen000039-Guh1] Guh A. Heyman M. L. Barden D. Fontana J. Hadler J. L. (2010). Lessons learned from the investigation of a cluster of cutaneous anthrax cases in Connecticut J Public Health Manag Pract 16 201–210 10.1097/PHH.0b013e3181ca650d . 20357605

[mgen000039-Keim1] Keim P. Price L. B. Klevytska A. M. Smith K. L. Schupp J. M. Okinaka R. Jackson P. J. Hugh-Jones M. E. (2000). Multiple-locus variable-number tandem repeat analysis reveals genetic relationships within *Bacillus anthracis* J Bacteriol 182 2928–2936 10.1128/JB.182.10.2928-2936.2000 . 10781564 PMC102004

[mgen000039-Koser1] Köser C. U. Holden M. T. G. Ellington M. J. Cartwright E. J. P. Brown N. M. Ogilvy-Stuart A. L. Hsu L. Y. Chewapreecha C. Croucher N. J. other authors (2012). Rapid whole-genome sequencing for investigation of a neonatal MRSA outbreak N Engl J Med 366 2267–2275 10.1056/NEJMoa1109910 . 22693998 PMC3715836

[mgen000039-Li1] Li H. Durbin R. (2009). Fast and accurate short read alignment with Burrows-Wheeler transform Bioinformatics 25 1754–1760 10.1093/bioinformatics/btp324 . 19451168 PMC2705234

[mgen000039-Li12] Li H. Handsaker B. Wysoker A. Fennell T. Ruan J. Homer N. Marth G. Abecasis G. Durbin R. 1000 Genome Project Data Processing Subgroup (2009). The Sequence Alignment/Map format and SAMtools Bioinformatics 25 2078–2079 10.1093/bioinformatics/btp352 . 19505943 PMC2723002

[mgen000039-Marston1] Marston C. K. Allen C. A. Beaudry J. Price E. P. Wolken S. R. Pearson T. Keim P. Hoffmaster A. R. (2011). Molecular epidemiology of anthrax cases associated with recreational use of animal hides and yarn in the United States PLoS One 6 e28274 10.1371/journal.pone.0028274 . 22174783 PMC3235112

[mgen000039-Nguyen1] Nguyen T. Q. Clark N. The 2006 NYC Anthrax Working Group (2010). Public health and environmental response to the first case of naturally acquired inhalational anthrax in the United States in 30 years: infection of a New York City resident who worked with dried animal hides J Public Health Manag Pract 16 189–200 10.1097/PHH.0b013e3181ca64f2 . 20357604

[mgen000039-Osborn1] Osborn S. H. (1920). Anthrax problem in Massachusetts Am J Public Health (N Y) 10 657–665 10.2105/AJPH.10.8.657 . 18010353 PMC1362846

[mgen000039-Pearson1] Pearson T. Okinaka R. T. Foster J. T. Keim P. (2009). Phylogenetic understanding of clonal populations in an era of whole genome sequencing Infect Genet Evol 9 1010–1019 10.1016/j.meegid.2009.05.014 . 19477301

[mgen000039-Price1] Price E. P. Seymour M. L. Sarovich D. S. Latham J. Wolken S. R. Mason J. Vincent G. Drees K. P. Beckstrom-Sternberg S. M. other authors (2012). Molecular epidemiologic investigation of an anthrax outbreak among heroin users, Europe Emerg Infect Dis 18 1307–1313 10.3201/eid1808.111343 . 22840345 PMC3414016

[mgen000039-Riley1] Riley A. (2007). Report on the management of an anthrax incident in the Scottish Borders, July 2006 to May 2007.http://news.bbc.co.uk/1/shared/bsp/hi/pdfs/13_12_07_anthrax.pdf.

[mgen000039-Rouli1] Rouli L. MBengue M. Robert C. Ndiaye M. La Scola B. Raoult D. (2014). Genomic analysis of three African strains of *Bacillus anthracis* demonstrates that they are part of the clonal expansion of an exclusively pathogenic bacterium New Microbes New Infect 2 161–169 10.1002/nmi2.62 . 25566394 PMC4265047

[mgen000039-Stratilo1] Stratilo C. Bader D. (2007). Molecular typing of *Bacillus anthracis* using multiple-locus variable number tandem repeat analysis (MLVA-8) Defence R&D Canada, Technical Memorandum. .

[mgen000039-Tamura1] Tamura K. Stecher G. Peterson D. Filipski A. Kumar S. (2013). mega6: Molecular Evolutionary Genetics Analysis version 6.0 Mol Biol Evol 30 2725–2729 10.1093/molbev/mst197 . 24132122 PMC3840312

[mgen000039-Treangen1] Treangen T. J. Ondov B. D. Koren S. Phillippy A. M. (2014). The Harvest suite for rapid core-genome alignment and visualization of thousands of intraspecific microbial genomes Genome Biol 15 524 10.1186/s13059-014-0524-x . 25410596 PMC4262987

[mgen000039-Van1] Van Ert M. N. Easterday W. R. Huynh L. Y. Okinaka R. T. Hugh-Jones M. E. Ravel J. Zanecki S. R. Pearson T. Simonson T. S. other authors (2007). Global genetic population structure of *Bacillus anthracis* PLoS One 2 e461 10.1371/journal.pone.0000461 . 17520020 PMC1866244

[mgen000039-Vogler1] Vogler A. J. Busch J. D. Percy-Fine S. Tipton-Hunton C. Smith K. L. Keim P. (2002). Molecular analysis of rifampin resistance in *Bacillus anthracis* and *Bacillus cereus* Antimicrob Agents Chemother 46 511–513 10.1128/AAC.46.2.511-513.2002 . 11796364 PMC127050

