# Supplementary material for: Whole-genome sequencing investigation of animal-skin-drum-associated UK anthrax cases reveals evidence of mixed populations and relatedness to a US case
Source: Microb Genom. 2015 Nov 7;1(5):e000039. doi: 10.1099/mgen.0.000039 (PMC5320680; doi:10.1099/mgen.0.000039)
Supplement: Supplementary file 1 — Supplementary Data [file mgen-01-39-s001.pdf]

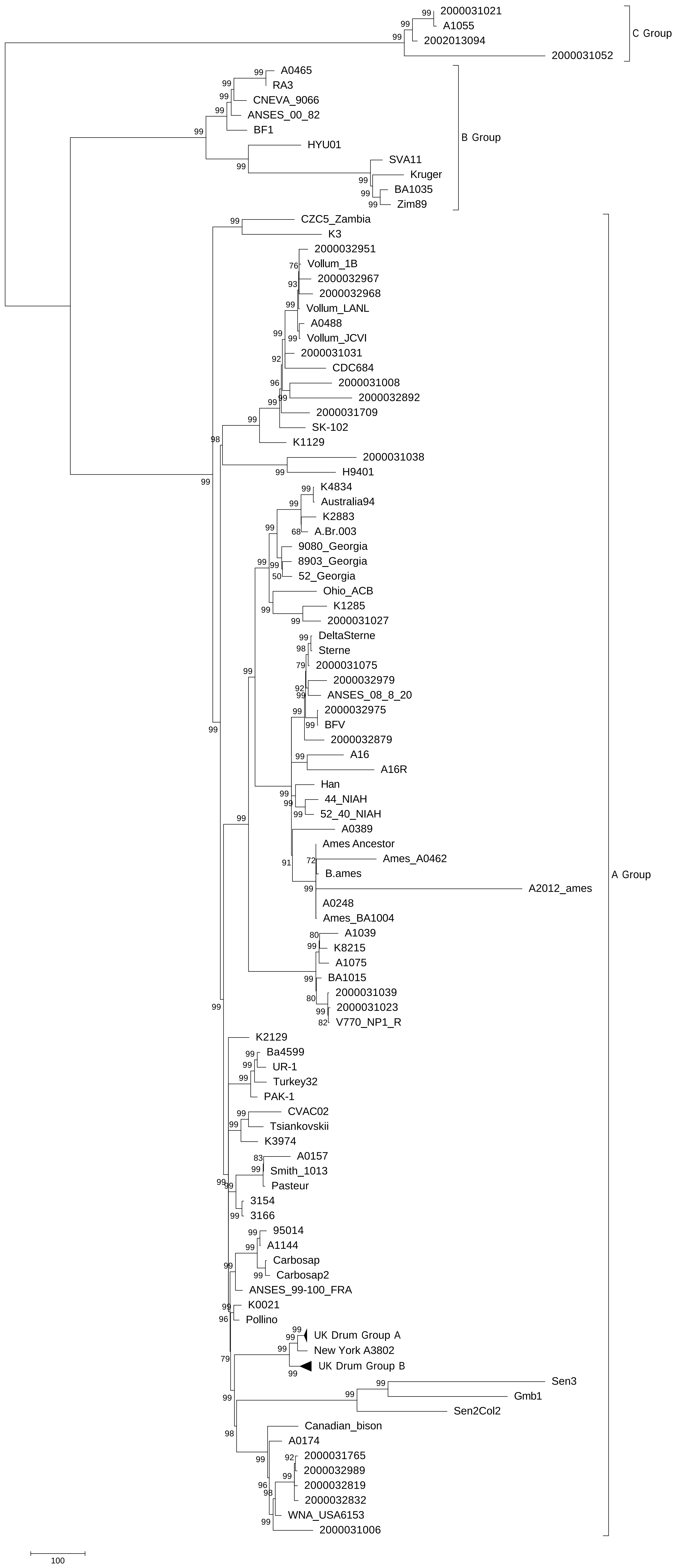
2000031021
A1055
2002013094
2000031052
C Group
A0465
RA3
CNEVA_9066
ANSES_00_82
BF1
HYU01
SVA11
Kruger
BA1035
Zim89
B Group
CZC5_Zambia
K3
2000032951
Vollum_1B
2000032967
2000032968
Vollum_LANL
A0488
Vollum_JCVI
2000031031
CDC684
2000031008
2000032892
2000031709
SK-102
K1129
2000031038
H9401
K4834
Australia94
K2883
A.Br.003
9080_Georgia
8903_Georgia
52_Georgia
Ohio_ACB
K1285
2000031027
DeltaSterne
Sterne
2000031075
2000032979
ANSES_08_8_20
2000032975
BFV
2000032879
A16
A16R
Han
44_NIAH
52_40_NIAH
A0389
Ames Ancestor
Ames_A0462
B.ames
A2012_ames
A0248
Ames_BA1004
A1039
K8215
A1075
BA1015
2000031039
2000031023
V770_NP1_R
K2129
Ba4599
UR-1
Turkey32
PAK-1
CVAC02
Tsiankovskii
K3974
A0157
Smith_1013
Pasteur
3154
3166
95014
A1144
Carbosap
Carbosap2
ANSES_99-100_FRA
K0021
Pollino
UK Drum Group A
New York A3802
UK Drum Group B
Sen3
Gmb1
Sen2Col2
Canadian_bison
A0174
2000031765
2000032989
2000032819
2000032832
WNA_USA6153
2000031006
A Group
100

Isolate	Profile	Sample source	vrrA	vrrB1	vrrB2	vrrC1	vrrC2	CG3	pX01	pX02
Scotland_459	M1	Environmental - Drum	313	229	153	468	498	153	120	135
Scotland_472	M1	Clinical	313	229	153	468	498	153	120	135
Scotland_473	M1	Clinical	313	229	153	468	498	153	120	135
Scotland_474	M2	Environmental - Hide	**289**	229	153	468	498	153	120	135
Scotland_476	M1	Environmental - Drum storage area	313	229	153	468	498	153	120	135
Scotland_477	M1	Environmental - Drum storage area	313	229	153	468	498	153	120	135
Scotland_478	M1	Environmental - Drum	313	229	153	468	498	153	120	135
Scotland_479	M1	Environmental - Drum playing area	313	229	153	468	498	153	120	135
Scotland_480	M1	Environmental - Drum playing area	313	229	153	468	498	153	120	135
Scotland_481	M1	Environmental - Drum playing area	313	229	153	468	498	153	120	135
Scotland_482	M1	Environmental - Drum playing area	313	229	153	468	498	153	120	135
Scotland_483	M1	Environmental - Drum storage area	313	-	153	468	498	153	120	135
Scotland_484	M1	Environmental - Drum storage area	313	229	153	468	498	153	120	135
Scotland_485	M1	Environmental - Drum storage area	313	-	153	468	498	153	120	135
London_491	M3	Clinical	313	229	162	512	498	153	123	135
London_493	M3	Environmental - Hide	313	-	162	512	498	-	-	135
London_494	M3	Environmental - Hide	313	229	162	512	498	153	-	135
London_496	M4	Environmental - Drum	**289**	229	162	**468**	498	153	123	135
London_497	M3	Environmental - Hide	313	229	162	512	498	153	123	135
London_498	M3	Environmental - Hide	313	229	162	512	498	-	-	135
London_499	M3	Environmental - Hide	313	229	-	512	498	153	123	135
London_500	M3	Environmental - Hide	313	229	-	512	498	153	123	135
London_501	M3	Environmental - Hide	313	229	-	512	498	153	-	135

Supplementary Table 1. Results of MLVA-8 analysis.

	Chr		pXO1		pXO2	
	Mean coverage	Stdev	Mean coverage	Stdev	Mean coverage	Stdev
**Scotland_459**	55.46	25.79	175.44	88.96	148.02	72.81
**Scotland_472**	64.61	27.67	218.12	105.9	146.72	69.77
**Scotland_473**	49.88	30.01	222.32	115.38	123.88	64.67
**Scotland_474**	47.62	22.24	293.37	139.14	138.33	63.01
**Scotland_476**	39.07	20.88	205.13	88.2	98.37	41.23
**Scotland_477**	43.09	21.31	233.67	118.79	125.55	62.73
**Scotland_478**	40	18.62	239.72	110.61	128.73	57.87
**Scotland_479**	41.03	18.7	229.89	105.55	121.98	54.22
**Scotland_480**	49.99	21.87	299.33	134.1	157.11	67.5
**Scotland_481**	60.03	31.31	282.56	158.49	164.38	89.92
**Scotland_482**	46.45	22.2	242.5	118.88	135.15	64.69
**Scotland_483**	70.96	35.89	417.36	191.56	221.74	99.93
**Scotland_484**	65.08	30.06	377.86	183.23	198.81	93.82
**Scotland_485**	95.75	44.14	535.33	268.9	298.04	144.05
**London_491**	52.61	23.25	171.37	83.77	111.99	51.98
**London_493**	69.37	287.5	147.33	76.28	82.68	41.13
**London_494**	48.63	25.16	259.74	134.84	122.43	61.26
**London_496**	49.91	24.05	284.28	129.54	1.12	6.29
**London_497**	61.51	28.51	369.75	184.23	161.77	75.86
**London_498**	56.45	26.77	332.46	161.6	1.36	7.57
**London_499**	53.78	23.1	325.3	143.17	119.81	52.19
**London_500**	48.94	24.45	259.41	122.33	137.52	63.85
**London_501**	53.98	26.5	299.52	144.74	1.25	6.83
**New York A3082**	254.69	58.11	432.56	49.3	386.57	77.44

Supplementary Table 2. Coverage data for each strain.

Name	Biosample	Bioproject	Size (Mb)	GC%	Scaffolds
3154	SAMN02470705	PRJNA178572	5.00269	35.40	3
3166	SAMN02470706	PRJNA178573	5.50514	35.20	5
95014	SAMN02951914	PRJNA224116	5.3368	35.20	27
2000031006	SAMN03165110	PRJNA224116	5.44337	35.10	75
2000031008	SAMN03165111	PRJNA224116	5.45101	35.10	37
2000031021	SAMN02736984	PRJNA224116	5.33174	35.36	-
2000031023	SAMN03165112	PRJNA224116	5.43554	35.10	33
2000031027	SAMN03165113	PRJNA224116	5.44555	35.10	51
2000031031	SAMN03165114	PRJNA224116	5.4493	35.10	44
2000031038	SAMN03165115	PRJNA224116	5.43107	35.10	48
2000031039	SAMN03165116	PRJNA224116	5.44962	35.10	34
2000031052	SAMN03165117	PRJNA224116	5.27409	35.20	54
2000031075	SAMN03165118	PRJNA224116	5.35337	35.10	46
2000031709	SAMN03165119	PRJNA224116	5.4496	35.10	35
2000031765	SAMN03165120	PRJNA224116	5.44942	35.10	43
2000032819	SAMN03165121	PRJNA224116	5.45031	35.10	45
2000032832	SAMN03165122	PRJNA224116	5.44926	35.10	36
2000032879	SAMN03165123	PRJNA224116	5.44748	35.10	53
2000032892	SAMN03165124	PRJNA224116	5.45053	35.10	42
2000032951	SAMN03165125	PRJNA224116	5.43193	35.10	56
2000032967	SAMN03165126	PRJNA224116	5.44337	35.10	62
2000032968	SAMN03165127	PRJNA224116	5.29679	35.10	90
2000032975	SAMN03165128	PRJNA224116	5.43915	35.10	51
2000032979	SAMN03165129	PRJNA224116	5.43321	35.10	54
2000032989	SAMN03165130	PRJNA224116	5.44396	35.10	53
2002013094	SAMN03174509	PRJNA224116	5.60108	35.27	-
44-NIAH	SAMD00026915	PRJDB3562	5.37514	35.10	124
52-40-NIAH	SAMD00026914	PRJDB3563	5.44982	35.10	36
52-G	SAMN02951870	PRJNA224116	5.50444	35.26	3
8903-G	SAMN02951868	PRJNA224116	5.50477	35.26	3
9080-G	SAMN02951869	PRJNA224116	5.50866	35.26	3
A.Br.003	SAMN02736983	PRJNA224116	5.48772	35.20	3
A0157	SAMN03267488	PRJNA224116	5.32224	35.46	2
A0174	SAMN02470265	PRJNA55003	5.29191	35.20	60
A0193	SAMN02470282	PRJNA55005	5.39288	35.10	60
A0248	SAMN02603932	PRJNA33543	5.50393	35.26	-
A0389	SAMN02470266	PRJNA27917	5.4204	35.17	68
A0442	SAMN02470279	PRJNA27915	5.37484	35.20	46
A0465	SAMN02470255	PRJNA27919	5.40715	35.20	57
A0488	SAMN02470254	PRJNA27913	5.39217	35.20	63
A1039	SAMN02999502	PRJNA224116	5.45826	35.10	32
A1055	SAMN02435882	PRJNA10795	5.37062	35.30	42
A1075	SAMN02999503	PRJNA224116	5.45987	35.07	26
A1144	SAMN02999504	PRJNA224116	5.47706	35.26	-
A16	SAMN02641483	PRJNA224116	5.5045	35.26	-
A16R	SAMN02641484	PRJNA224116	5.40945	35.30	-
A2012	SAMN02435829	PRJNA54101	5.37006	35.16	3

Ames	SAMN02603432	PRJNA57909	5.22729	35.40	-
Ames A0462	SAMN03290655	PRJNA224116	5.50391	35.26	-
Ames Ancestor; A2084	SAMN02603433	PRJNA10784	5.50393	35.26	-
Ames_BA1004	SAMN03201418	PRJNA224116	5.50397	35.26	-
ANSES_00-82	SAMN02699416	PRJNA224116	5.43601	35.10	28
ANSES_08-8_20	SAMN02693047	PRJNA224116	5.44071	35.10	31
ANSES_99-100	SAMN02699415	PRJNA224116	5.44647	35.10	30
Australia 94	SAMN02435830	PRJNA10799	5.50456	35.20	49
BA1015	SAMN03010426	PRJNA224116	5.49118	35.26	-
BA1035	SAMN03010427	PRJNA224116	5.48726	35.26	-
BF1	SAMN02469407	PRJNA171093	5.43453	35.06	56
BFV	SAMN02736972	PRJNA224116	5.50835	35.27	3
Canadian_bison	SAMN03202901	PRJNA224116	5.50577	35.26	-
Carbosap	SAMN02910129	PRJNA224116	5.40767	35.10	18
Carbosap	SAMN02470598	PRJNA198410	5.40297	35.10	21
CDC 684	SAMN02603931	PRJNA31329	5.50676	35.26	-
CNEVA-9066	SAMN02435891	PRJNA10796	5.48868	35.20	30
Cvac02	SAMN02898388	PRJNA224116	5.22717	35.40	-
CZC5	-	PRJNA224116	5.45434	35.10	25
delta Sterne	SAMN02736981	PRJNA224116	5.22965	35.40	1
Gmb1	SAMEA2272630	PRJNA198415	5.17508	35.20	39
H9401	SAMN02603474	PRJNA49361	5.49547	35.26	-
Han	SAMN02898318	PRJNA224116	5.22543	35.40	-
Heroin Ba4599	SAMN02470707	PRJNA72893	5.46838	35.10	46
HYU01	SAMN02874036	PRJNA224116	5.49012	35.26	-
K0021	SAMN03757457	PRJNA257008	5.45962	35.10	31
K1129	SAMN03757460	PRJNA257008	5.46076	35.10	36
K1285	SAMN03757454	PRJNA257008	5.46083	35.10	36
K2129	SAMN03757455	PRJNA257008	5.45942	35.10	28
K2883	SAMN03757456	PRJNA257008	5.45845	35.10	32
K3	SAMN03010428	PRJNA224116	5.50499	35.26	-
K3974	SAMN03757459	PRJNA257008	5.45099	35.10	41
K4834	SAMN03757458	PRJNA257008	5.45061	35.10	69
K8215	SAMN03757461	PRJNA257008	5.45873	35.10	42
Kruger B	SAMN02435884	PRJNA324	5.47001	35.10	64
Ohio ACB	SAMN03010429	PRJNA224116	5.49834	35.26	-
PAK-1	SAMN03010430	PRJNA224116	5.40338	35.30	-
Pasteur	SAMN03024436	PRJNA224116	5.2948	35.36	-
Pollino	SAMN03296000	PRJNA224116	5.50389	35.26	3
RA3	SAMN03075602	PRJNA224116	5.48987	35.26	-
Sen2Col2	SAMEA2272511	PRJNA198413	5.17411	35.20	44
Sen3	SAMEA2272292	PRJNA198414	5.17379	35.20	50
SK-102	SAMN03012770	PRJNA224116	5.50566	35.27	-
Smith 1013	SAMN02732407	PRJNA224116	5.28699	35.26	2
Sterne	SAMN02598266	PRJNA58091	5.22866	35.40	-
SVA11	SAMN03081486	PRJNA224116	5.48752	35.26	-

Tsiankovskii-I	SAMN02436236	PRJNA17709	5.50473	35.20	56
Turkey32	SAMN03010432	PRJNA224116	5.5053	35.26	-
UR-1	SAMN02469406	PRJNA170826	5.44661	35.10	109
V770-NP-1R	SAMN03092715	PRJNA224116	5.4104	35.30	-
Vollum	SAMN02736982	PRJNA224116	5.50619	35.26	-
Vollum	SAMN02435883	PRJNA10797	5.48846	35.20	52
Vollum 1B	SAMN03010433	PRJNA224116	5.50663	35.26	-
Western North America USA6153	SAMN02435885	PRJNA337	5.51135	35.20	44
Zimbabwe 89	SAMN02736952	PRJNA224116	5.46092	35.10	3
London_491	SAMN03790801	PRJNA287512	5.65748	35.13	61
London_493	SAMN03790802	PRJNA287512	5.43900	35.09	38
London_494	SAMN03790803	PRJNA287512	5.48906	35.14	126
London_496	SAMN03790804	PRJNA287512	5.43824	35.11	77
London_497	SAMN03790805	PRJNA287512	5.66571	35.12	90
London_498	SAMN03790806	PRJNA287512	5.43545	35.09	88
London_499	SAMN03790807	PRJNA287512	5.59241	35.13	87
London_500	SAMN03790808	PRJNA287512	5.65717	35.13	51
London_501	SAMN03790809	PRJNA287512	5.44360	35.10	39
Scotland_459	SAMN03790787	PRJNA287512	5.43102	35.10	85
Scotland_472	SAMN03790788	PRJNA287512	5.55289	35.12	55
Scotland_473	SAMN03790789	PRJNA287512	5.43875	35.10	50
Scotland_474	SAMN03790790	PRJNA287512	5.43733	35.09	36
Scotland_476	SAMN03790791	PRJNA287512	5.44180	35.09	33
Scotland_477	SAMN03790792	PRJNA287512	5.53287	35.12	75
Scotland_478	SAMN03790793	PRJNA287512	5.52391	35.23	149
Scotland_479	SAMN03790794	PRJNA287512	5.65273	35.14	95
Scotland_480	SAMN03790795	PRJNA287512	5.34597	35.13	47
Scotland_481	SAMN03790796	PRJNA287512	5.43893	35.11	85
Scotland_482	SAMN03790797	PRJNA287512	5.34147	35.13	57
Scotland_483	SAMN03790798	PRJNA287512	5.43958	35.08	33
Scotland_484	SAMN03790799	PRJNA287512	5.44473	35.11	58
Scotland_485	SAMN03790800	PRJNA287512	5.34105	35.12	35
New York A3802		PRJNA287512	5.44514	35.08	26

Supplementray Table 3. Sequences used in phylogenetic analysis.
